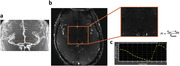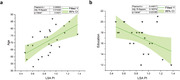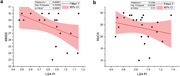# Association of pulsatility of lenticulostriate arteries with cognitive decline in elderly adults: a 7T dual‐VENC PC‐MRI study

**DOI:** 10.1002/alz.093252

**Published:** 2025-01-09

**Authors:** Jianing Tang, Tianrui Zhao, Elizabeth B Joe, Helena C Chui, Lirong Yan

**Affiliations:** ^1^ Department of Radiology, Northwestern University, Chicago, IL USA; ^2^ Department of Neurology, Keck School of Medicine, University of Southern California, Los Angeles, CA USA; ^3^ Stevens Neuroimaging and Informatics Institute, University of Southern California, Los Angeles, CA USA

## Abstract

**Background:**

Cerebral small vessel disease (cSVD) is a major cause of vascular dementia. Mounting evidence suggests that cSVD pathology is closely linked to vascular dysfunction of lenticulostriate arteries (LSAs). Arterial pulsatility, quantified by pulsatility index (PI), has been used as an indicator of vascular dysfunction. With the advent of ultra‐high field 7T, the velocity and pulsatility of LSAs can be reliably measured. Directly assessing pulsatility of LSAs may offer valuable insight into pathophysiology of cSVD and cognitive impairment. This study aims to investigate relationship between LSA pulsatility and cognitive performance within an aged cohort.

**Method:**

Twenty‐five elderly participants (13 female: 71 ± 9.2 years) were enrolled in the study with written informed consent. Cognitive assessments were conducted including 19 participants undergoing the Mini‐Mental State Exam (MMSE) and 23 taking Montreal Cognitive Assessment (MoCA). High‐resolution phase‐contrast MRI (PC‐MRI) with dual‐VENC (Venc=20cm/s and 40cm/s) was performed on a Siemens 7T MRI scanner to image velocities of LSAs. PI was calculated by the difference between the peak systolic flow velocity and minimum diastolic flow velocity divided by the mean velocity throughout a cardiac cycle. Pearson correlation was used to evaluate the associations between LSA PI and age, education level, and cognitive measurements. A linear mixed‐effects model was used to test the association between LSA PI and cognitive measurements while correcting for the effects of age, gender, and education.

**Result:**

Flow velocity curves and PI values of LSAs were successfully extracted and calculated from all 25 participants. Figure 1 shows an example of LSA imaging using PC‐MRI and the acquired velocity curve of LSAs. LSA PI significantly increased with age and lower education levels (p=0.002, 0.03, respectively) (Figure 2). Increased PI was strongly associated with lower MMSE scores with and without corrections for age, gender, and educational level (p=0.048, 0.028) (Figure 3). A similar trend was observed between PI and MOCA scores, although there was no significance (p=0.31).

**Conclusion:**

This study has demonstrated that LSA pulsatility assessed by 7T high‐resolution PC‐MRI is strongly associated with aging, education, and cognitive performance. These findings suggest the dysfunction of LSA could contribute to the cSVD pathology and result in cognitive impairment.